# A role of TRPA1 in mechanical hyperalgesia is revealed by pharmacological inhibition

**DOI:** 10.1186/1744-8069-3-40

**Published:** 2007-12-17

**Authors:** Matt Petrus, Andrea M Peier, Michael Bandell, Sun Wook Hwang, Truc Huynh, Nicholas Olney, Tim Jegla, Ardem Patapoutian

**Affiliations:** 1 Genomics Institute of the Novartis Research Foundation, San Diego, CA 92121, USA; 2 Department of Cell Biology, The Scripps Research Institute, La Jolla, CA 92037, USA; 3 Merck Research Laboratories, Rahway, NJ, USA; 4 College of Medicine, Korea University, Seoul, South Korea

## Abstract

Mechanical hyperalgesia is a clinically-relevant form of pain sensitization that develops through largely unknown mechanisms. TRPA1, a Transient Receptor Potential ion channel, is a sensor of pungent chemicals that may play a role in acute noxious mechanosensation and cold thermosensation. We have developed a specific small molecule TRPA1 inhibitor (AP18) that can reduce cinnameldehyde-induced nociception *in vivo*. Interestingly, AP18 is capable of reversing CFA-induced mechanical hyperalgesia in mice. Although TRPA1-deficient mice develop normal CFA-induced hyperalgeisa, AP18 is ineffective in the knockout mice, consistent with an on-target mechanism. Therefore, TRPA1 plays a role in sensitization of nociception, and that compensation in TRPA1-deficient mice masks this requirement.

## Background

Sensory neurons of the dorsal root ganglia (DRGs) can detect environmental changes through projections in the skin. Among these primary afferent fibers, nociceptors play an important role in sensing noxious levels of mechanical and thermal stimuli [1]. One of the basic properties of nociceptors is their sensitization in response to injury and inflammation. For example, an innocuous stimulus such as a warm shower can become painful after sunburn, and this is called allodynia. Indeed, cold or mechanical allodynia are serious medical problems for patients suffering from neuropathic pain [2,3]. Hyperalgesia describes a similar condition, defined as an increased response to an already painful stimulus due to injury or inflammation.

TRPV1, a member of the Transient Receptor Potential family of cation channels, is activated by heat, low pH, and capsaicin [4]. TRPV1 is expressed in nociceptors, and is sensitized in response to a variety of signal transduction pathways activated during inflammation [5,6]. TRPV1-deficient mice display negligible inflammatory heat hyperalgesia [7,8]. Much less is known about molecules involved in mechanical hyperalgesia.

Originally characterized as a noxious cold-activated ion channel, TRPA1 is expressed in the same sensory neurons as TRPV1, and is activated directly by diverse reactive chemicals via covalent modification, and indirectly through G-protein coupled receptors [9-12]. TRPA1 is required to sense these noxious reactive chemicals *in vivo*; however, whether TRPA1 is required to sense acute noxious cold and mechanical stimulus is not yet settled [13-17]. Here, we examine the consequences of acute block of peripheral TRPA1 for pain transduction, focusing on mechanical hyperalgesia.

## Results

**Figure 1:**
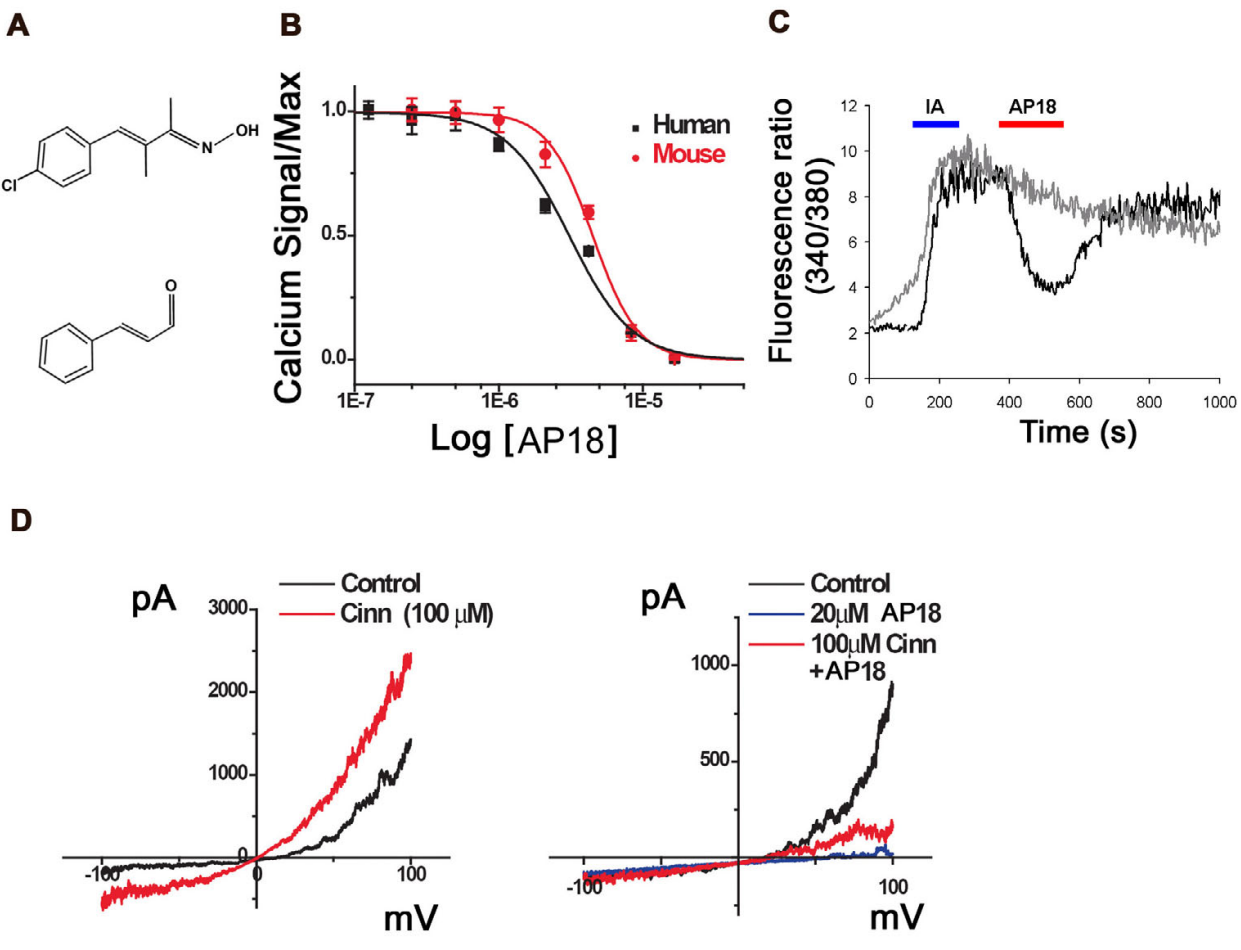
AP18 blocks TRPA1 activation. (**A**) Chemical structures of the AP18 (upper) and cinnamaldehyde (lower). (**B**) Dose-response relationships for block of the calcium influx by AP18 into CHO cells expressing mouse and human TRPA1 elicited by 50 μM cinnamaldehyde (left panel). Calcium influx was measured using a standard FLIPR assay, data points are the average of four wells (~8,000 cells/well) and error bars show standard error. Values are normalized to the maximal response (observed in the absence of AP18). (**C**) Ratiometric Ca^2+ ^imaging of average of ~50 mTRPA1-expressing CHO cells in response to 1 mM Iodoacetamide (IA) and 100 μM of AP18 (black trace). Grey trace represents cells that were treated with IA alone. (**D**) Current-voltage relationship of TRPA1. Outward rectifying currents elicited by cinnamaldehyde (left panel) in inside-out macropatches derived from TRPA1-expressing *Xenopus *oocytes were suppressed by AP18 coapplications (right panel).

**Figure 2:**
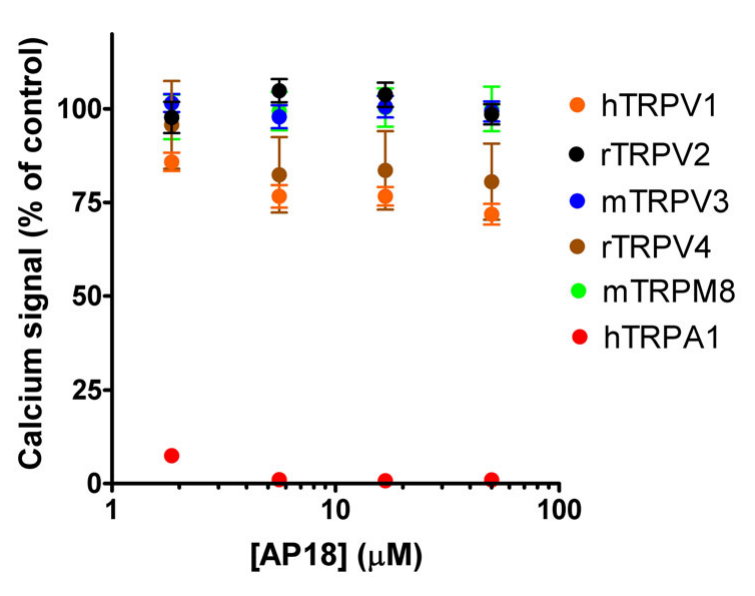
**AP18 specifically blocks TRPA1**. Calcium influx (FLIPR) in HEK cells transiently transfected with the indicated cDNAs in response to 200 nM capsaicin (TRPV1), 150 μM 2-APB (TRPV2), 30 μM 2-APB (TRPV3), 20 μM 4alpha-PDD (TRPV4), 20 μM menthol (TRPM8), and 80 μM cinnamaldehyde (TRPA1). Values are given as a percentage of agonist response in the absence of AP18.

**Figure 3:**
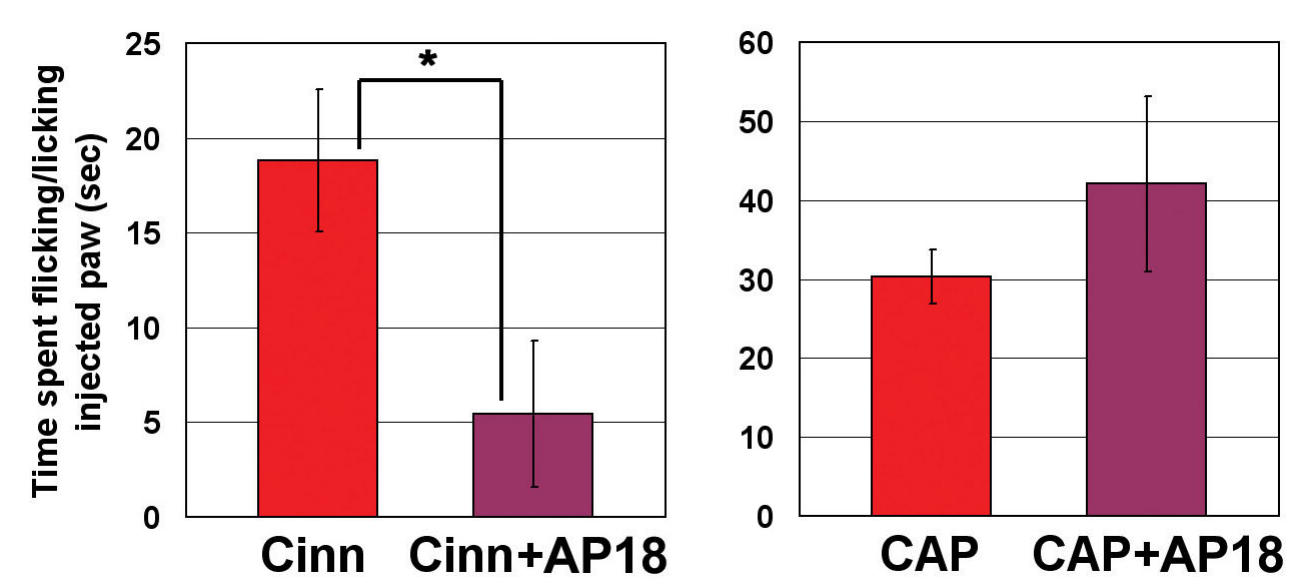
AP18 suppresses acute nociceptive behaviours caused by cinnamaldehyde but not capsaicin. Time spent licking and flicking hindpaws injected with cinnamaldehyde (16.4 mM) or capsaicin (0.328 mM) was measured for five minutes and compared with animals co-injected with AP18 (1 mM). Numbers of cases for each experiment from the left is 8, 8, 6 and 6, respectively (*p < 0.05, two-tailed Student's *t*-test).

To test if acute block of TRPA1 can modulate pain sensation, we sought a specific, efficient TRPA1 inhibitor. Ruthenium red, gadolinium, menthol, and camphor can inhibit TRPA1 activation, but also interact with other channels, including other TRP channels[18]. Using a Fluorometric Imaging Plate Reader (FLIPR, Molecular Devices) calcium-influx assay, we screened 43,648 small molecules for their ability to block cinnamaldehyde-activation of human TRPA1 in a Chinese Hamster Ovary (CHO) cell line. We further characterized one of these hits, AP18 ((Z)-4-(4-chlorophynyl)-3-methylbut-3-en-2-oxime – Maybridge, Cornwall, UK; Figure 1A). AP18 blocked activation of TRPA1 by 50 μM cinnamaldehyde with an IC_50 _of 3.1 μM and 4.5 μM for human and mouse clones, respectively (Figure 1B). At concentrations up to 50 μM, AP18 was unable to appreciably block activation of TRPV1, TRPV2, TRPV3, TRPV4, or TRPM8 (Figure 2). AP18 reversibly blocked mouse TRPA1 responses to iodoacetamide (an irreversible cysteine-alkylating agent) in CHO cells assayed by ratiometric Ca^2+ ^imaging (Figure 1C). AP18 also blocked cold- and mustard-oil-induced activation of mouse TRPA1 (data not shown). Moreover, AP18 blocked cinnamaldehyde-induced TRPA1 currents in excised patches from *Xenopus *oocytes (Figure 1D). *In vivo*, 1–10 mM of AP18 injected in hindpaw of mice did not cause any obvious behavioural responses (data not shown). Importantly, AP18 significantly blocked cinnamaldehdye-induced but not capsaicin-induced nociceptive events, demonstrating efficacy and specificity (Figure 3).

**Figure 4:**
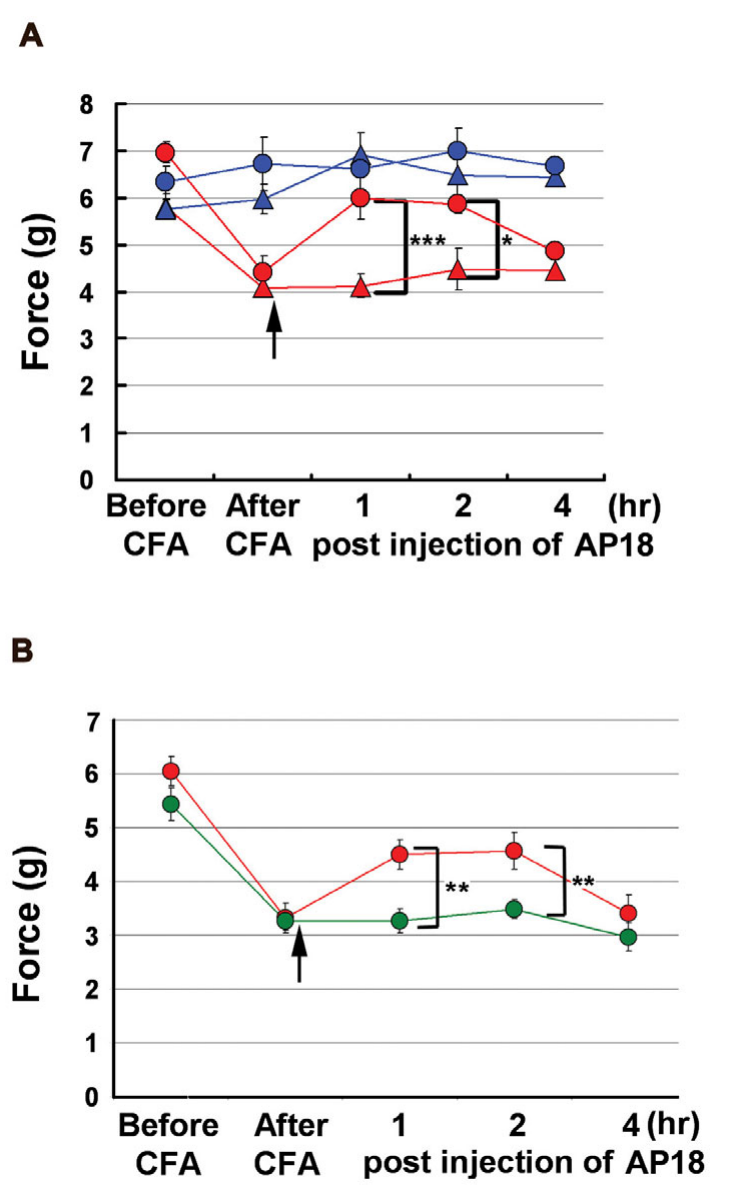
TRPA1 is involved in CFA-induced mechanical hyperalgesia. (**A**) AP18 reverses CFA-induced mechanical hyperalgseia in wildtype mice (n = 8). Red symbols represent the paws treated with CFA. 24 hours later, mechanical sensitivity was assayed again ("after CFA" timepoint), and the same paws were injected with either AP18 (red circles) or vehicle (red triangle). Mechanical sensitivity was again monitored during the next four hours. Blue symbols represent the control uninjected paws. (**B**) AP18 reverses mechanical hyperalgesia in wildtype (red) but not TRPA1-deficient (green) littermates (n = 12, females). Same protocol as (A), data from CFA- and AP18-injected paws shown. Arrows indicate time of AP18 (or vehicle) injections. ***p < 0.001, **p < 0.01, *p < 0.05, two-tailed Student's *t*-test.

**Figure 5:**
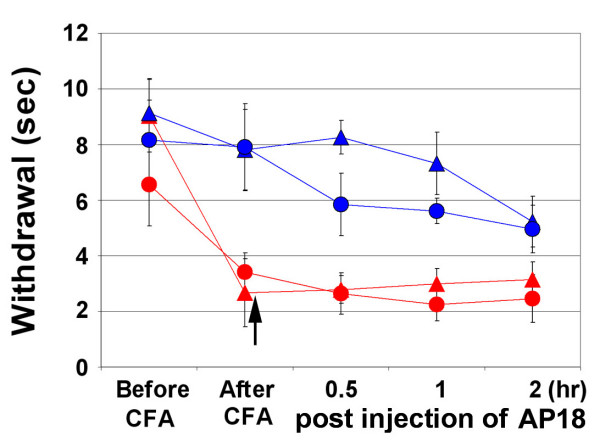
AP18 does not reverse CFA-induced heat hyperalgesia. Same designation of symbols as in Figure 4A is used. Arrow indicates time of AP18 (or vehicle) injections. Paw-withdrawal latencies to infrared 20 setting are measured and averaged (n = 8).

**Figure 6:**
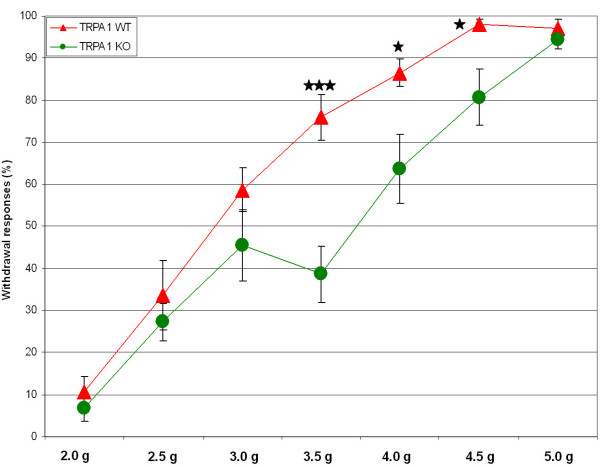
Acute mechanosensory phenotype in TRPA1-deficient mice. Frequency of paw withdrawal to fixed forces applied to the hindpaw is measured. 13 WT and 11 TRPA1-deficient male mice were tested. Briefly, hindpaws were exposed to the force actuator starting with the greatest force. Each force was applied eight times at ~1/s. The actual g-force values used differ between this study and Kwan et al. (2006) probably due to the use of different instrumentation [16].

**Figure 7:**
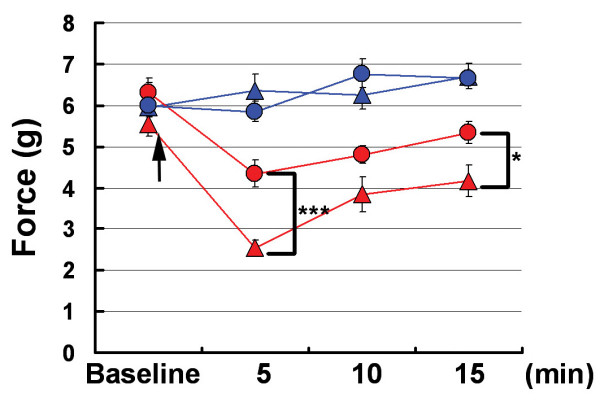
Bradykinin (BK)-induced mechanical hyperalgesia is partially blocked by AP18. Same designation of symbols as in Figure 4A is used; however, BK (instead of CFA) is co-injected with AP18 in wildtype mice, and mechanical sensitivity is measured within 15 minutes (n = 12). Arrow indicates time of AP18 (or vehicle) injections. ***p < 0.001, *p < 0.05, two-tailed Student's *t*-test.

Sensitization of nociceptive neurons is the basis for many chronic pain states [1]. To investigate the role of TRPA1 in pain sensitization, we tested AP18 in mechanical hyperalgesia induced by complete Freund's adjuvant (CFA). Mechanical sensitivity was measured using an automated Von Frey apparatus which records the point at which the paw is withdrawn in response to a progressively-increasing force. AP18 but not vehicle solution injected in the paw 24 hours after CFA treatment almost completely reversed mechanical hyperalgesia (Figure 4A). AP18 did not reverse CFA-induced heat hyperalgesia (Figure 5). Acute nociceptive responses to both increasing mechanical force and infrared noxious heat applications to the paw without coincident inflammation were not affected by AP18 treatment (data not shown). Remarkably, AP18 did not reverse CFA-induced mechanical hyperalgesia in TRPA1-deficient mice, suggesting on-target mechanisms for the activity of AP18 (Figure 4B) [16,17]. It is important to note that prior to AP18 treatment, TRPA1-deficient mice developed normal CFA-induced mechanical hyperalgesia (Figure 4B), suggesting that compensation in these mice masks the requirement for TRPA1 in mechanical sensitization (see discussion below). Also note that TRPA1-deficient mice do not show significantly increased acute mechanical threshold before CFA application in this assay (Figure 4B). However, we do observe the previously-reported deficit in acute mechanosensation in TRPA1-deficient mice at higher g-forces when using the protocol of Kwan et al.(2006) which assays frequency of paw withdrawal in response to a fixed (rather than increasing) force applied to the paw (Figure 6) [16]. In addition to the acute mechanosensory phenotype, TRPA1-deficient mice have strong deficits in bradykinin-induced inflammatory pain [16,17]. As expected, AP18-treated mice showed reduced bradykinin-induced mechanical hyperalgesia compared to vehicle (Figure 7).

**Figure 8:**
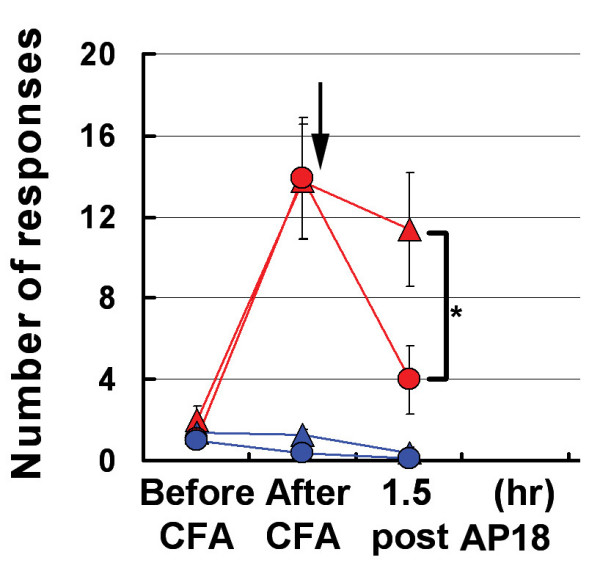
AP18 partially reverses cold hyperalgesia in rats treated with CFA. Same designation of symbols as Figure 4A is used. Number of flicks, licks, paw raises are counted for 10 minutes and averaged on a 5°C cold plate (n = 8). Arrow indicates time of AP18 (or vehicle) injections. *p < 0.05, two-tailed Student's *t*-test.

Whether TRPA1 is activated by noxious cold temperatures, and whether TRPA1-deficient mice show deficits in cold thermosensation is unsettled, and area of active research [9,16,17,19-22]. We have found it difficult to demonstrate noxious cold response in wildtype mice using temperatures as low as 0°C, either alone or following CFA injection (data not shown). Sprague Dawley rats, on the other hand, readily display cold hyperalgesia, and TRPA1 is implicated in this process [21]. CFA-induced cold hyperalgesia assayed on a 5°C plate is significantly reversed in rats treated with AP18, supporting a role of TRPA1 in cold nociception (Figure 8).

## Discussion

The consequence of acute pharmacological block of protein activity in adult animals sometimes deviates from the effects of germline ablation of the encoding gene. For example, it was recently shown that pharmacological inhibition of TRPV1 induces hyperthermia in wildtype mice in a TRPV1-dependent manner, although mice born genetically-deficient in TRPV1 do not display a hyperthermic phenotype [23,24]. Using a small molecule inhibitor of TRPA1 we have demonstrated a novel role of TRPA1 in mechanical hyperalgesia. AP18 is ineffective in reversing CFA-induced mechanical hyperalgesia in TRPA1-deficient mice, suggesting an on-target mechanism for AP18. What compensatory mechanisms may account for the normal mechanical hyperalgesia observed in TRPA1-deficient mice? Upregulation of another protein that plays a similar role as TRPA1 (molecular compensation) is one explanation. TRPA1 is the only member of the TRPA family in mammals, and TRPV1, another nocisensor expressed in pain-sensing neurons, does not appear to be upregulated in TRPA1-deficient mice, although the two channels functionally interact [16,17,25]. TRPV4, a related ion channel, has been implicated in some forms of mechanical hyperalgesia and can be an intriguing candidate [26]. However, whether TRPV4 expression is required in skin cells or DRGs is unsettled [27]. Cellular compensation (i.e. a change in the composition of neuronal subtypes or in neuronal connectivity) is another potential explanation for the normal hyperalgesia observed in TRPA1-deficient mice. Furthermore, it is possible that TRPA1 is involved in the maintenance of mechanical hyperalgesia rather than its induction. In this case, normal development of mechanical hyperalgesia is expected in TRPA1-deficient mice, but compensation could contribute to maintenance of the condition.

TRPA1 is specifically expressed in nociceptors, its transcripts are upregulated in response to CFA in rats, and inflammatory signals sensitize the channel, consistent with TRPA1 being involved in hyperalgesia [21,28,29]. The mechanism by which TRPA1 contributes specifically to mechanosensation is less clear [30]. It is possible that mammalian TRPA1 is directly activated by mechanical forces, although no such data is currently present. Alternatively, mechanical damage could indirectly activate TRPA1 through release of reactive compounds or intracellular calcium (both of which can directly activate mammalian TRPA1) [11,12,31]. One other possibility is that TRPA1 is involved in modulation of mechanosensory signaling, and not directly involved with mechanotransduction. Interestingly, the ortholog of mammalian TRPA1 in *C. elegans *can be activated by pressure applied through a recording pipette when expressed in heterologous cells (although it is also not clear if cTRPA1 is directly or indirectly responding to this mechanical force) and plays an essential role in mechanosensory behaviour [32].

In conclusion, this study highlights a novel role of mouse TRPA1 in mechanical hyperalgesia, and underscores the importance of considering both acute pharmacological block and genetic ablations in assigning gene function.

## Methods

### FLIPR Screen and Calcium imaging

CHO cells expressing human TRPA1 were plated into 384-well plates at a concentration of ~8,000 cells/well. Cells were transferred to phosphate-buffered saline (PBS) and loaded with the calcium sensitive dye FLUO-4 using the FLIPR Calcium 3 Assay Kit (Molecular Devices, Sunnyvale, CA) one hour prior to the assay. Assays were run using the FLIPR2 (Molecular Devices, Sunnyvale, CA). All compounds were diluted into PBS from a high concentration DMSO-based stock solution and added during data collection with the FLIPR2 internal pipette head. Final DMSO concentrations never exceeded 0.5%. Ratiometric Ca^2+ ^imaging on tet-inducible mouse TRPA1 in CHO cells was performed as described[9].

### *Xenopus *oocyte excised patches

Human TRPA1 was cloned into the pOX expression vector and cRNA transcripts were produced using the T3 mMessage Machine kit (Ambion, TX)[33]. Mature defolliculated *Xenopus *oocytes were injected with 50 nL of human TRPA1 cRNA at ~1 μg/μL. Oocytes were incubated in ND96 (96 mM NaCl, 2 mM KCl, 1 mM MgCl_2_, 1.8 mM CaCl_2_, 5 mM HEPES, pH 7.4, supplemented with Na-pyruvate (2.5 mM), penicillin (100 u/mL) and streptomycin (100 μg/mL) for three to five days to ensure sufficient TRPA1 expression. Vitelline envelopes were mechanically removed prior to experiments. Recordings were made under voltage clamp from excised patches in the inside-out configuration at room temperature with 1–1.5 MΩ pipettes. The bath ground was isolated using an agar bridge. Capacitance and series resistance were compensated and solutions that eliminate native calcium-activated chloride currents were used (Patch electrode (in mM): 140 NaMES, 4 NaCl, 1 EGTA, 10 HEPES, pH 7.2; bath solution: 140 KMES, 4 KCl, 1 EGTA, 10 HEPES, pH 7.2). Compounds were added to the bath solution. Currents were recorded using a Multiclamp 700B amplifier and the pCLAMP acquisition suite.

### Behavioral assays

Mice of 6–16 weeks in age and 150–250 g Sprague Dawley rats were used for all behavioural assays. C57BL6/J mice were used, except in Figure 3b: TRPA1-deficient mice and WT littermates were on mixed genetic background [16]. Animals were acclimated for 20–60 min to their testing environment prior to all experiments. Student's *t- *test was used for all statistical calculations. All error bars represent standard error of the mean (SEM). Hargreaves apparatus (Plantar Analgesia meter) and Von Frey apparatus (Dynamic Plantar Aesthesiometer) are from UGO Basile. Mechanical or thermal hyperalgesia assays were performed as described [7,34]. Briefly, mice were acclimated for 60 minutes to the testing environment prior to all experiments. Baseline responses were measured first and then 10 nM BK in 20 μl was injected to the skin of left hindpaws. Von Frey threshold or paw withdrawal latency was measured at 5, 15 and 30 minutes post injection. 1 mM of AP18 was coinjected to left hindpaws to test its analgesic effect. For CFA-induced hyperalgesia testing, 10 μg CFA in 10 μL was injected into mice and 50 μg in 100 μL (1:1 emulsion of mineral oil and saline) was injected into rats and 24-hour measurements were performed [7,21,35]. 1 mM of AP18 was injected in 10 μl. Control and AP18 solutions were in PBS, 0.5% Tween80 and 1% DMSO. Before the measurements, animals were re-acclimated to the environment for 20–60 minutes. Different time points were used for experiments with CFA-injected animals (30 min, 1, 1 1/2, 2 and 4 hr after AP18 injection).

### Chemicals

All chemicals were purchased from Sigma-Aldrich unless otherwise described. Capsaicin was purchased from Fluka.

## References

[B1] Woolf CJ, Ma Q (2007). Nociceptors-noxious stimulus detectors. Neuron.

[B2] Ossipov MH, Lai J, Malan TP, Porreca F (2000). Spinal and supraspinal mechanisms of neuropathic pain. Ann N Y Acad Sci.

[B3] Woolf CJ (2004). Dissecting out mechanisms responsible for peripheral neuropathic pain: implications for diagnosis and therapy. Life Sci.

[B4] Caterina MJ, Schumacher MA, Tominaga M, Rosen TA, Levine JD, Julius D (1997). The capsaicin receptor: a heat-activated ion channel in the pain pathway. Nature.

[B5] Bhave G, Gereau RW (2004). Posttranslational mechanisms of peripheral sensitization. J Neurobiol.

[B6] Hucho T, Levine JD (2007). Signaling pathways in sensitization: toward a nociceptor cell biology. Neuron.

[B7] Caterina MJ, Leffler A, Malmberg AB, Martin WJ, Trafton J, Petersen-Zeitz KR, Koltzenburg M, Basbaum AI, Julius D (2000). Impaired nociception and pain sensation in mice lacking the capsaicin receptor. Science.

[B8] Davis JB, Gray J, Gunthorpe MJ, Hatcher JP, Davey PT, Overend P, Harries MH, Latcham J, Clapham C, Atkinson K, Hughes SA, Rance K, Grau E, Harper AJ, Pugh PL, Rogers DC, Bingham S, Randall A, Sheardown SA (2000). Vanilloid receptor-1 is essential for inflammatory thermal hyperalgesia. Nature.

[B9] Story GM, Peier AM, Reeve AJ, Eid SR, Mosbacher J, Hricik TR, Earley TJ, Hergarden AC, Andersson DA, Hwang SW, McIntyre P, Jegla T, Bevan S, Patapoutian A (2003). ANKTM1, a TRP-like Channel Expressed in Nociceptive Neurons, Is Activated by Cold Temperatures. Cell.

[B10] Bandell M, Story GM, Hwang SW, Viswanath V, Eid SR, Petrus MJ, Earley TJ, Patapoutian A (2004). Noxious Cold Ion Channel TRPA1 Is Activated by Pungent Compounds and Bradykinin. Neuron.

[B11] Macpherson LJ, Dubin AE, Evans MJ, Marr F, Schultz PG, Cravatt BF, Patapoutian A (2007). Noxious compounds activate TRPA1 ion channels through covalent modification of cysteines. Nature.

[B12] Hinman A, Chuang HH, Bautista DM, Julius D (2006). TRP channel activation by reversible covalent modification. Proc Natl Acad Sci U S A.

[B13] Trevisani M, Siemens J, Materazzi S, Bautista DM, Nassini R, Campi B, Imamachi N, Andre E, Patacchini R, Cottrell GS, Gatti R, Basbaum AI, Bunnett NW, Julius D, Geppetti P (2007). 4-Hydroxynonenal, an endogenous aldehyde, causes pain and neurogenic inflammation through activation of the irritant receptor TRPA1. Proc Natl Acad Sci U S A.

[B14] McNamara CR, Mandel-Brehm J, Bautista DM, Siemens J, Deranian KL, Zhao M, Hayward NJ, Chong JA, Julius D, Moran MM, Fanger CM (2007). TRPA1 mediates formalin-induced pain. Proc Natl Acad Sci U S A.

[B15] Macpherson LJ, Xiao B, Kwan KY, Petrus MJ, Dubin AE, Hwang S, Cravatt B, Corey DP, Patapoutian A (2007). An ion channel essential for sensing chemical damage. J Neurosci.

[B16] Kwan KY (2006). TRPA1 contributes to cold, mechanical, and chemical nociception but is not essential for hair-cell transduction. Neuron.

[B17] Bautista DM, Jordt SE, Nikai T, Tsuruda PR, Read AJ, Poblete J, Yamoah EN, Basbaum AI, Julius D (2006). TRPA1 Mediates the Inflammatory Actions of Environmental Irritants and Proalgesic Agents. Cell.

[B18] Macpherson LJ, Hwang SW, Miyamoto T, Dubin AE, Patapoutian A, Story GM (2006). More than cool: Promiscuous relationships of menthol and other sensory compounds. Mol Cell Neurosci.

[B19] Sawada Y, Hosokawa H, Hori A, Matsumura K, Kobayashi S (2007). Cold sensitivity of recombinant TRPA1 channels. Brain Res.

[B20] Dhaka A, Viswanath V, Patapoutian A (2006). TRP Ion Channels and Temperature Sensation. Annu Rev Neurosci.

[B21] Obata K, Katsura H, Mizushima T, Yamanaka H, Kobayashi K, Dai Y, Fukuoka T, Tokunaga A, Tominaga M, Noguchi K (2005). TRPA1 induced in sensory neurons contributes to cold hyperalgesia after inflammation and nerve injury. J Clin Invest.

[B22] Jordt SE, Bautista DM, Chuang HH, McKemy DD, Zygmunt PM, Hogestatt ED, Meng ID, Julius D (2004). Mustard oils and cannabinoids excite sensory nerve fibres through the TRP channel ANKTM1. Nature.

[B23] Steiner AA, Turek VF, Almeida MC, Burmeister JJ, Oliveira DL, Roberts JL, Bannon AW, Norman MH, Louis JC, Treanor JJ, Gavva NR, Romanovsky AA (2007). Nonthermal activation of transient receptor potential vanilloid-1 channels in abdominal viscera tonically inhibits autonomic cold-defense effectors. J Neurosci.

[B24] Gavva NR, Bannon AW, Surapaneni S, Hovland DN, Lehto SG, Gore A, Juan T, Deng H, Han B, Klionsky L, Kuang R, Le A, Tamir R, Wang J, Youngblood B, Zhu D, Norman MH, Magal E, Treanor JJ, Louis JC (2007). The vanilloid receptor TRPV1 is tonically activated in vivo and involved in body temperature regulation. J Neurosci.

[B25] Ruparel NB, Patwardhan AM, Akopian AN, Hargreaves KM (2007). Homologous and heterologous desensitization of capsaicin and mustard oil responses utilize different cellular pathways in nociceptors. Pain.

[B26] Alessandri-Haber N, Dina OA, Joseph EK, Reichling D, Levine JD (2006). A transient receptor potential vanilloid 4-dependent mechanism of hyperalgesia is engaged by concerted action of inflammatory mediators. J Neurosci.

[B27] Chung MK, Lee H, Mizuno A, Suzuki M, Caterina MJ (2004). TRPV3 and TRPV4 mediate warmth-evoked currents in primary mouse keratinocytes. J Biol Chem.

[B28] Diogenes A, Akopian AN, Hargreaves KM (2007). NGF up-regulates TRPA1: implications for orofacial pain. J Dent Res.

[B29] Dai Y, Wang S, Tominaga M, Yamamoto S, Fukuoka T, Higashi T, Kobayashi K, Obata K, Yamanaka H, Noguchi K (2007). Sensitization of TRPA1 by PAR2 contributes to the sensation of inflammatory pain. J Clin Invest.

[B30] Christensen AP, Corey DP (2007). TRP channels in mechanosensation: direct or indirect activation?. Nat Rev Neurosci.

[B31] Zurborg S, Yurgionas B, Jira JA, Caspani O, Heppenstall PA (2007). Direct activation of the ion channel TRPA1 by Ca(2+). Nat Neurosci.

[B32] Kindt KS, Viswanath V, Macpherson L, Quast K, Hu H, Patapoutian A, Schafer WR (2007). Caenorhabditis elegans TRPA-1 functions in mechanosensation. Nat Neurosci.

[B33] Jegla T, Salkoff L (1997). A novel subunit for shal K+ channels radically alters activation and inactivation. J Neurosci.

[B34] Moqrich A, Hwang SW, Earley TJ, Petrus MJ, Murray AN, Spencer KS, Andahazy M, Story GM, Patapoutian A (2005). Impaired thermosensation in mice lacking TRPV3, a heat and camphor sensor in the skin. Science.

[B35] Cao YQ, Mantyh PW, Carlson EJ, Gillespie AM, Epstein CJ, Basbaum AI (1998). Primary afferent tachykinins are required to experience moderate to intense pain. Nature.

